# Hemodynamic consequences of TEVAR with different configurations of *in-situ* fenestration stents: a patient-specific CFD analysis

**DOI:** 10.3389/fcvm.2026.1824395

**Published:** 2026-04-29

**Authors:** Yunpeng Ding, Songjie Hu, Di Wang, Dehai Lang, Tiequan Yang, Chunbo Xu

**Affiliations:** 1 Department of Vascular Surgery, Ningbo No.2 Hospital, Wenzhou Medical University, Ningbo, China; 2 Department of Interventional Radiology, Ningbo No.2 Hospital, Wenzhou Medical University, Ningbo, China

**Keywords:** computational fluid dynamics, fenestrated stent, haemodynamics, ISF-TEVAR, left subclavian revascularization

## Abstract

**Objectives:**

This exploratory study aimed to utilize patient-specific computational fluid dynamics (CFD) simulations to evaluate and compare the hemodynamic effects of different fenestration stent configurations following *in situ* fenestration thoracic endovascular aortic repair (ISF-TEVAR).

**Methods:**

Six patient-specific aortic models (pre- and postoperative) were reconstructed from CTA images of three ISF-TEVAR patients with distinct fenestration stent configurations. The hemodynamic parameters of the aortic arch and its main branches (including pressure, flow velocity, flow volume, and vascular wall shear stress (WSS)) were systematically analyzed to evaluate the effects of the stent configuration.

**Results:**

Marked increases in pressure drop and WSS were observed in the left subclavian artery (LSA) in cases with higher stent compression. Compared to forward-leaning stent orientation, a near-perpendicular insertion was associated with more pronounced complex and disordered flow patterns around the fenestration site.

**Conclusion:**

In this small case series, different fenestration stent configurations significantly influenced local hemodynamics, particularly in the LSA. Minimizing stent compression and, where anatomically feasible, adopting a forward-leaning orientation may help reduce adverse hemodynamic effects. These preliminary findings highlight the potential utility of patient-specific CFD simulation in preoperative planning for ISF-TEVAR, though further studies with larger cohorts are needed to validate these observations.

## Introduction

Thoracic endovascular aortic repair (TEVAR) was currently the preferred treatment option for thoracic aortic diseases (1). However, approximately 40% of thoracic aortic lesions involve the left subclavian artery (LSA), making it impossible to treat them using conventional TEVAR (2). Although occluding the LSA can provide sufficient anchoring area for the stent, this operation significantly increases the risks of stroke and spinal ischemia, among others (3). Therefore, it is generally recommended to perform LSA revascularization concurrently during TEVAR to maintain blood supply to its branches (4, 5). Standard ISF-TEVAR maintains perfusion of aortic branches with the use of some tools to open the hole and place a stent (6, 7). Since the first report of successful clinical application of ISF to reconstruct the left subclavian artery (LSA) to repair thoracic aortic aneurysm, *in situ* fenestration technique have been increasingly used for LSA revascularization (8). Its postoperative complication rate was low, and *in situ* fenestration technique proved to be a promising treatment for aortic branch revascularization (9). However, stroke and aortic branch occlusion is still the main problems of ISF-TEVAR (10). Although TEVAR perioperative and postoperative stroke rates are low in previous reports (11). The occurrence of in-stent thrombosis may be associated with local hemodynamic disturbances induced by specific stent structures, which not only promote thrombus formation but may also compromise distal blood perfusion (12). Quantitative assessment of postoperative hemodynamic parameters facilitates a more accurate understanding of the efficacy of ISF-TEVAR and provides a basis for optimizing surgical strategies. However, traditional clinical monitoring methods struggle to comprehensively and precisely capture intravascular hemodynamic details. In recent years, computational fluid dynamics (CFD) technology has increasingly become a vital tool for simulating and analyzing blood flow characteristics (13). The application of CFD to aortic pathology typically involves a multi-step workflow: from patient-specific CTA data, three-dimensional anatomical models are reconstructed via image segmentation and surface meshing; computational meshes are then generated, followed by definition of boundary conditions (e.g., inlet flow waveforms and outlet pressure models); finally, the Navier-Stokes equations are solved to simulate blood flow and derive hemodynamic parameters including pressure, velocity, and wall shear stress. Validation studies have demonstrated that CFD can accurately replicate clinical observations. For instance, Polanczyk et al. (14) reported that CFD matched Doppler ultrasound with 99% accuracy in post-TEVAR aortic dissection patients. Furthermore, recent frameworks have integrated structural analysis of stent-graft deployment with fluid-dynamics simulations to predict post-TEVAR hemodynamics from pre-operative images. Romarowski et al. (15) introduced a distance-image based method enabling preoperative identification of high-risk features such as the bird-beak configuration, thus supporting more comprehensive planning. For example, Kandail et al. previously applied CFD to evaluate the hemodynamic performance of different open-window stents in abdominal aortic aneurysm repair (16). Nevertheless, specific hemodynamic studies in patients undergoing left subclavian artery fenestration during TEVAR remain limited at present.

Therefore, this study was designed to systematically evaluate the hemodynamic effects of left subclavian artery fenestration following ISF-TEVAR by establishing patient-specific CFD models, aiming to provide theoretical guidance for clinical practice and device design.

## Materials and methods

### Patient selection

From 2020 to 2021, we reviewed three ISF-TEVAR cases for aortic disease.This study included 3 patients who underwent ISF-TEVAR treatment, all of whom provided informed consent. Case 1 involved an 84-year-old male diagnosed with a penetrating aortic ulcer. Case 2 and Case 3 involved a 65-year-old male and a 50-year-old male, respectively, both diagnosed with aortic dissection. All patients had lesions involving the LSA. To achieve adequate proximal anchorage and restore LSA perfusion, all underwent ISF-TEVAR.

### Intravascular intervention techniques

The analysis of primary aortic lesions was performed by two specialists with over 10 years of experience in preoperative assessment. The selected stents were oversized by no more than 10%, and the final stent deployment site was determined based on intraoperative aortic angiography. A 6F catheter sheath was placed via percutaneous puncture of the left brachial artery (LBA). Subsequently, a 5F angiographic catheter was advanced under guidewire guidance to the ascending aorta for angiography. Simultaneously, the femoral artery was exposed. After catheter placement in the ascending aorta, the guidewire was replaced with a super-stiff guidewire, and the main stent was delivered to the aortic arch along this guidewire. Ankura stents (Shenzhen Xianjian Technology) were used as the main stents in all three patients in this group. The main stent was deployed when systolic blood pressure dropped below 90 mmHg. Subsequently, a puncture needle was used to create an opening in the main stent, through which a guidewire was advanced into the newly established window. A balloon catheter was then advanced over the guidewire. A balloon with a diameter of 4–8 mm was selected to pre-dilate the window until the “gourd-shaped” stenotic morphology was eliminated. Finally, a covered stent graft Viabahn (W. L. Gore & Associates, Flagstaff, USA) was deployed into the lumen via the left subclavian artery (11 × 50 mm for Cases 1 and 3; 13 × 50 mm for Case 2). Subsequently, a post-dilation of the stented segment was performed using an angioplasty balloon (8–12 mm diameter) matched to the diameter of the window stent. Immediately after the procedure, an aortic angiogram was performed to confirm the absence of internal leakage and unobstructed blood flow in the arch branches.

### Stent specifications

The main stent graft used in all three cases was the Ankura stent (Shenzhen Xianjian Technology, China), a self-expanding device composed of a nitinol (nickel-titanium alloy) framework with a polyester (PET) covering. Nitinol provides superelasticity and shape memory, enabling the stent to self-expand to its pre-set diameter upon deployment while maintaining conformability to the aortic arch anatomy. The fenestration branch stent deployed into the left subclavian artery was the Viabahn covered stent (W. L. Gore & Associates, Flagstaff, USA), also a self-expanding device. The Viabahn consists of an expanded polytetrafluoroethylene (ePTFE) lining with an outer fluorinated ethylene propylene (FEP) coating and a nitinol helical support structure, designed to provide flexibility, kink resistance, and a smooth luminal surface. In all cases, post-dilation was performed using angioplasty balloons (8–12 mm diameter) to ensure full expansion and optimal apposition at the fenestration interface.

### Postoperative follow-up and imaging evaluation

Regular annual follow-up visits are conducted for all patients postoperatively to monitor changes in symptoms and physical signs. Abdominal CTA images are acquired one year following ISF-TEV AR repair to construct three-dimensional models. Additionally, two physicians jointly evaluate preoperative and postoperative CTA images, focusing on aortic morphological changes, the presence of endoleaks, and stent positioning.

### Quantification of stent configuration

The insertion angle (*θ*) of the windowed stent relative to the lumen centerline of the main stent graft was measured on the postoperative 3D model. Case 1 demonstrated an anteverted angle (*θ* = 30°), while Cases 2 and 3 exhibited near-vertical insertion angles (*θ* = 85–90°). The compression ratio was calculated as (1 – (minimum cross-sectional area/nominal stent cross-sectional area)) × 100%.

### Mesh generation and CFD simulations

Based on the patient's raw CTA DICOM data, a three-dimensional aortic model was reconstructed using Mimics 21.0 software (Materialise, Belgium). To simplify computations, three aortic arch branches were maintained (brachiocephalic trunk, left common carotid artery, and left subclavian artery), while all other descending aortic branches were excluded. All inlet and outlet cross-sections underwent planar clipping in GeoMagic Studio (GeoMagic Inc, USA) to facilitate boundary condition definition.

**Figure 1:**
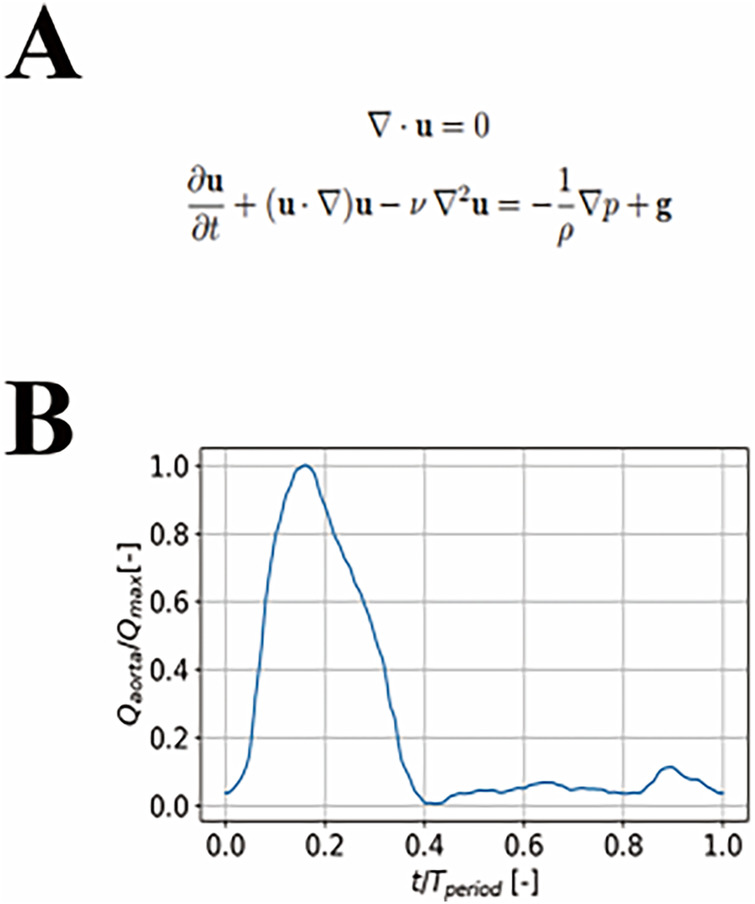
**(A)** the navier-stokes (NS) equations. **(B)** ascending aorta blood flow waveform.

The hemodynamic calculations were conducted on the established three-dimensional model. A computational fluid dynamics (CFD) solver based on the finite volume method (Boea Wisdom, Hangzhou) was employed to solve the three-dimensional transient Navier-Stokes equations (Figure 1A). Blood was modeled with incompressible Newtonian fluid properties, with a density of 1,060 kg/m³ and a dynamic viscosity of 0.004 Pa·s (17). This assumption is widely accepted for CFD simulations in large arteries (diameter > 1 mm) under physiological flow conditions, where shear rates exceed 100 s⁻¹ and non-Newtonian effects become negligible (18, 19), primarily due to red blood cell aggregation and deformation. Comparative studies have demonstrated that the Newtonian assumption yields acceptable accuracy for velocity profiles and wall shear stress predictions in the aorta, with differences relative to non-Newtonian models typically below 10% (20). Moreover, the use of a consistent fluid model across all cases preserves the validity of the relative hemodynamic comparisons that form the basis of our analysis. The blood flow was assumed to be laminar, as the Reynolds number in the aortic arch and its major branches during peak systole was estimated to range between 2,000 and 4,500, indicating transitional rather than fully complex and disordered flow patterns. This laminar assumption has been widely adopted in comparative patient-specific CFD studies of thoracic endovascular repair and is considered appropriate for evaluating relative hemodynamic differences across configurations (14, 16). Consequently, the three-dimensional, unsteady Navier–Stokes equations were solved without incorporating an additional turbulence model. Blood vessels are modeled as rigid walls with no slip. Each outlet is coupled to a three-element Windkessel model (WK3) (21), calibrated using the patient's measured systolic and diastolic blood pressures and estimated branch flow proportions. Patient-specific inflow velocity waveforms were not available for the retrospective cases included in this study. Therefore, a representative ascending aortic flow waveform from the literature (22) was scaled to match each patient's clinically measured cardiac output, heart rate, and systole/diastole duration ratio (Figure 1B). This approach has been widely employed in patient-specific CFD studies of the thoracic aorta and TEVAR, as it provides physiologically realistic inflow conditions while preserving patient-specific flow rates (12, 22). The spatial discretization of the governing equations employs a second-order upwind scheme, while the temporal discretization uses a first-order implicit Euler scheme.

The computational mesh was generated using ANSYS ICEM CFD (ANSYS, Inc., Canonsburg, PA, USA). Tetrahedral elements were employed due to their flexibility in conforming to the complex patient-specific geometry, particularly around the fenestration interface where intricate flow patterns were expected. Local mesh refinement was applied in regions of anticipated high flow gradients, including the fenestration site, the stent–stent junction, and the LSA ostium, with element sizes as small as 0.05 mm in these refined zones. A mesh independence study was performed to ensure that the numerical results were not sensitive to mesh density. Three mesh configurations were evaluated, with total element counts of approximately 25.2 million, 48.7 million, and 72.65 million elements. The peak systolic velocity and peak WSS in the LSA were selected as key parameters for comparison. The differences between the medium (48.7 million) and fine (72.65 million) meshes were less than 2% for both parameters, indicating mesh independence. The fine mesh (72.65 million elements) was selected for all subsequent simulations to ensure optimal resolution of flow details while maintaining computational efficiency. Mesh quality was assessed using skewness and aspect ratio metrics; the average skewness was maintained below 0.85, the average aspect ratio below 8, and the minimum orthogonal quality above 0.15, consistent with established guidelines for cardiovascular CFD simulations (23).

The time step was set to 10 ms, validated through time-step independence verification. The convergence tolerance was set to 10⁻⁵. The simulation ran continuously for three cardiac cycles to achieve periodic steady state, with the peak systolic value from the final cycle selected for subsequent analysis.

Numerical validation was performed through mesh independence and time-step independence studies as described above. Clinical validation was supported by the consistency of simulated hemodynamic patterns with postoperative CTA findings, which confirmed stent patency and the absence of flow-limiting stenoses in all cases. Furthermore, the simulated pressure and flow distributions in the aortic arch branches fell within expected physiological ranges reported in the literature for similar patient populations (12, 22). Although direct comparison with *in vivo* flow measurements (e.g., Doppler ultrasound) was not available for these retrospective cases, the combination of numerical verification and physiological consistency supports the credibility of the model for comparative hemodynamic assessment across stent configurations.

The covered stents used in the cases included in this study varied in diameter (Case 2: 13 mm; Case 1 & 3: 11 mm). To avoid misleading direct comparisons of absolute flow velocities due to differing inherent cross-sectional areas of the stents, this study focused on analyzing the distribution patterns of blood flow velocity, levels of turbulent kinetic energy, and relative changes between preoperative and postoperative states across cases when examining flow velocity-related parameters. By comparing the trends in flow velocity changes before and after surgery in the same patient, combined with a qualitative assessment of flow disturbance using flow field visualization (e.g., streamlines, vorticity maps), the study aimed to more accurately reveal the substantive impact of stent placement on local blood flow patterns, rather than relying solely on peak flow velocity for cross-case comparisons.

## Results

### Clinical outcomes

The surgical procedures for all three cases were successful, with no adverse events such as bleeding, stroke, paraplegia, infection, or death during the perioperative period. All patients received standard dual antiplatelet therapy (aspirin plus clopidogrel) for at least six months postoperatively, followed by lifelong single antiplatelet therapy.

At one-year follow-up, CTA imaging demonstrated patent LSA stents in all three cases, with no evidence of in-stent thrombosis, significant stenosis, or endoleak. No stent displacement was observed. Clinically, all patients remained asymptomatic, with no signs of upper limb ischemia, vertebrobasilar insufficiency, or other neurological deficits.

Despite the varying degrees of stent compression (Case 1: 11%, Case 2: 45%, Case 3: 58%) and the adverse hemodynamic profiles predicted by CFD in Cases 2 and 3, no patient developed clinical or imaging evidence of LSA insufficiency or thrombosis during the one-year follow-up period.

### Different configurations of fenestration stent

**Figure 2:**
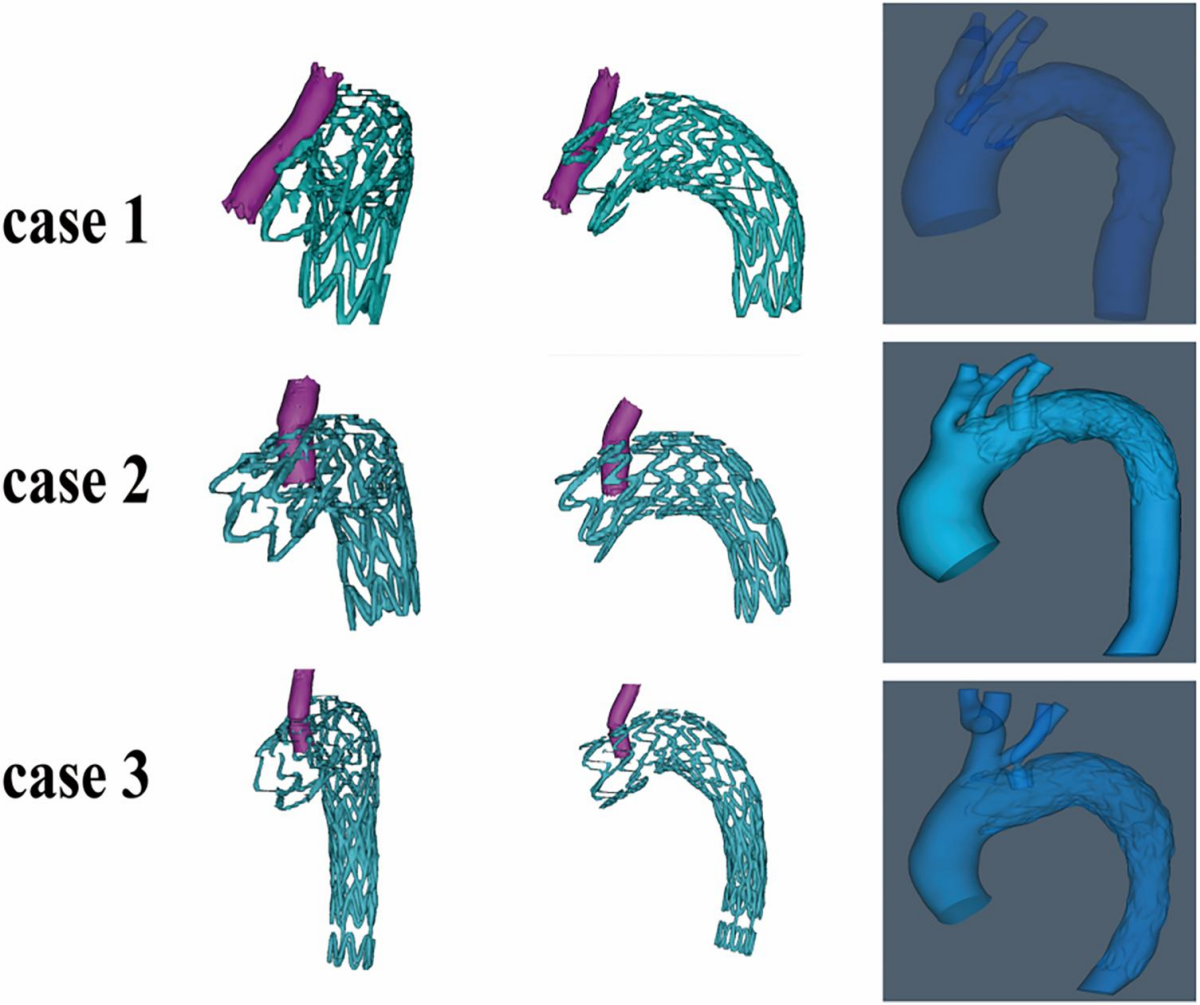
The 3D model of cases.

As illustrated in Figure 2, the fenestration stent configurations varied among the three cases. In Case 1, the fenestration stent was inserted into the main stent with a forward-leaning orientation (*θ* = 30°) and a compression ratio of 11%. In Cases 2 and 3, the fenestration stent was inserted in a near-vertical orientation (*θ* = 85–90°), with compression ratios of 45% and 58%, respectively.

### Pressure analyses

**Figure 3:**
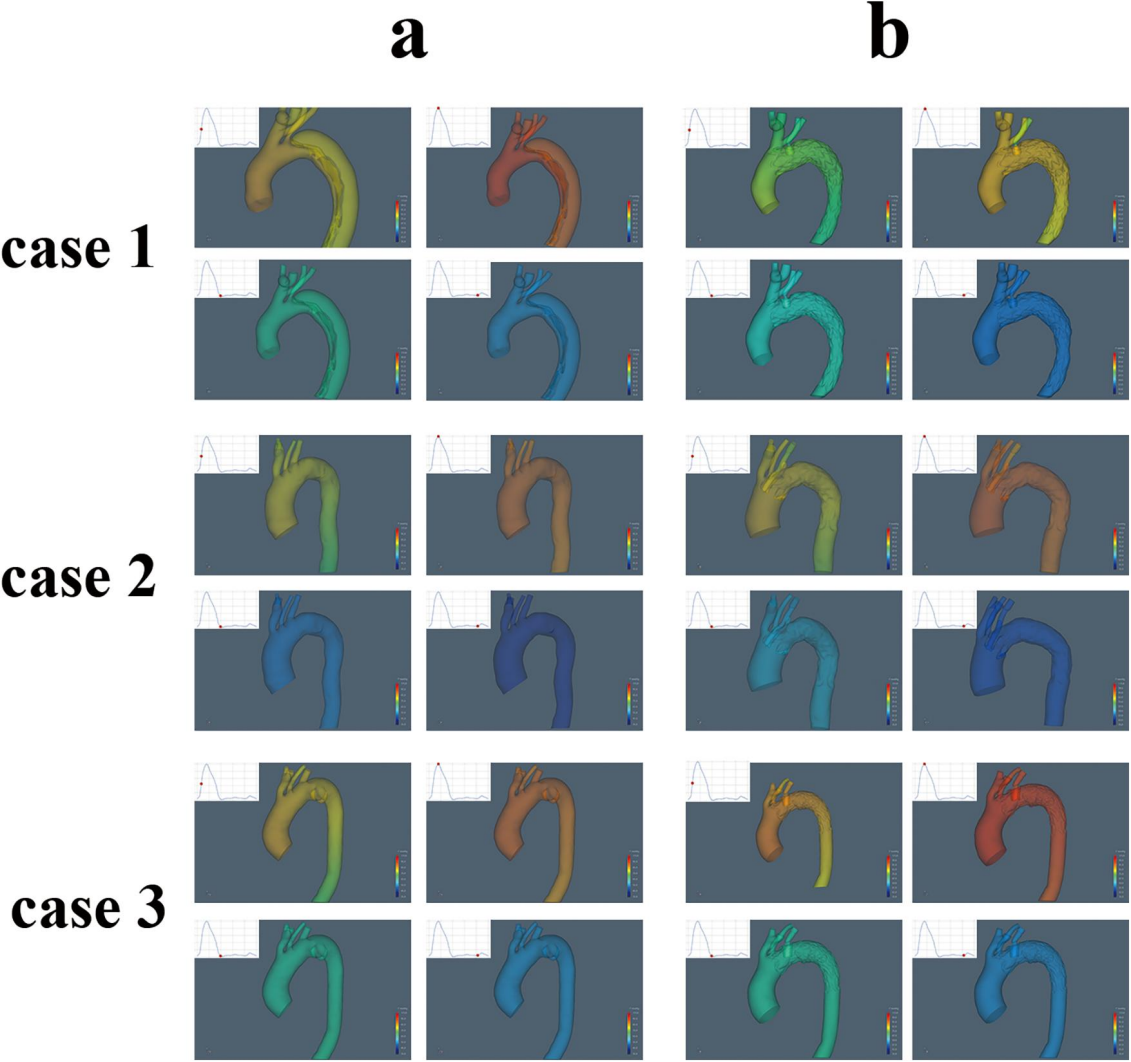
The preoperative and postoperative blood pressure profiles in the aortic arch and its major branches throughout a cardiac cycle (a: pre-operation, b: post-operation).

**Figure 4:**
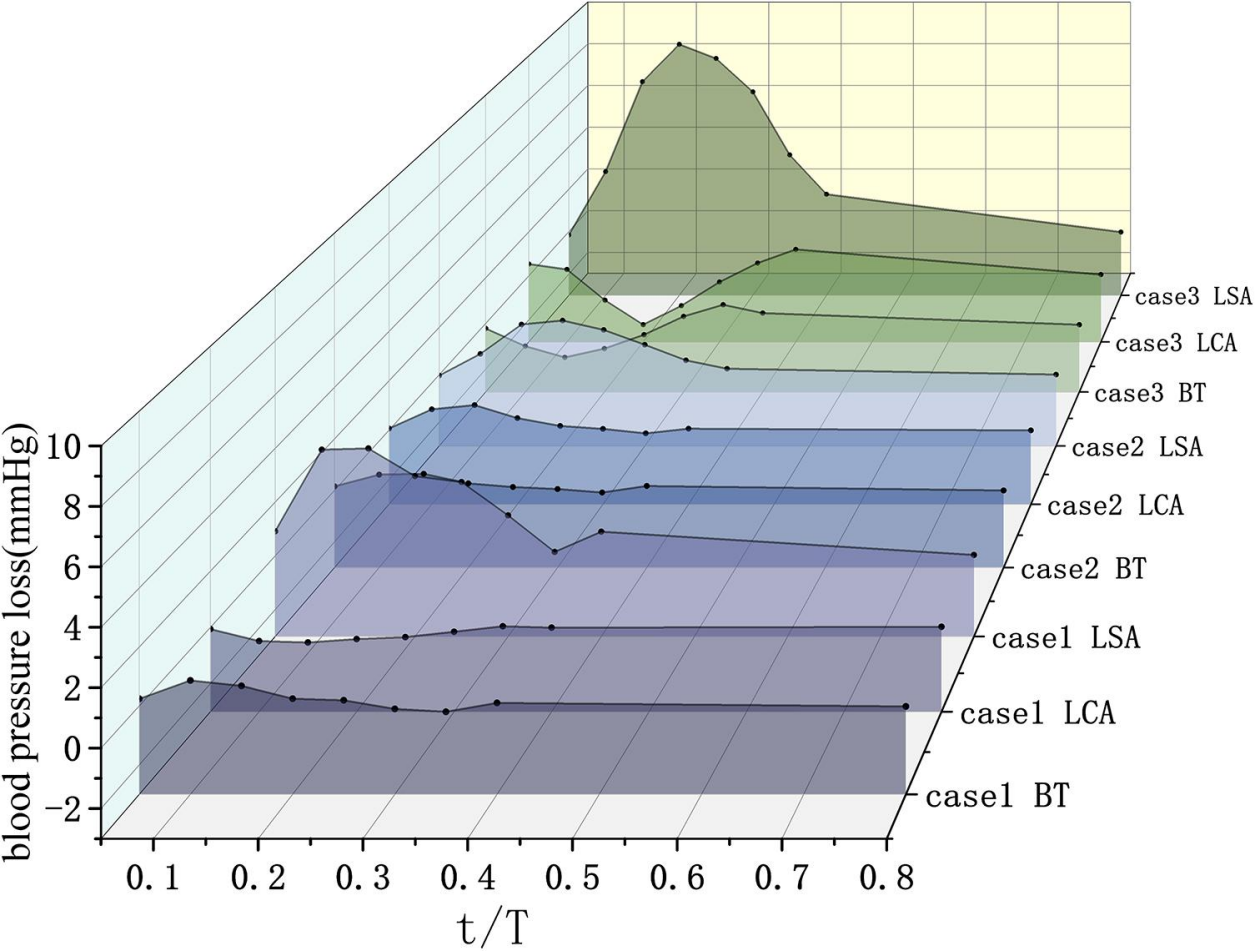
The pressure drop of the major branches of the aortic arch during various periods of the cardiac cycle (BT, brachiocephalic trunk; LCA, left carotid artery; LSA, left subclavian artery).

The preoperative and postoperative blood pressure profiles in the aortic arch and its major branches throughout a cardiac cycle are shown in Figure 3. Following ISF-TEVAR, a decrease in systolic blood pressure was observed in the LSA. The pressure drop was calculated as the difference between preoperative and postoperative pressures. As detailed in Table 1 and visualized in Figure 4, which depicts the pressure loss in the brachiocephalic trunk, left common carotid artery, and LSA, the systolic pressure loss in the LSA varied among cases. Case 3 exhibited the greatest systolic pressure loss (7.06 mmHg), followed by Case 1 (4.00 mmHg), while Case 2 demonstrated the smallest loss (2.12 mmHg). In contrast, pressure changes in the brachiocephalic trunk and left common carotid artery were minimal across all cases.

**Table 1:** Comparison of key hemodynamic parameters at peak-systole and mid-diastole among the three cases with different stent configurations.

Parameter	Case 1 (Forward tilt, Compression 11%)	Case 2 (Vertical, Compression 45%)	Case 3 (Vertical, Compression 58%)
A. Peak-Systole (15% of cardiac cycle)			
LSA Pressure (mmHg)*			
Preoperative	95.890	97.680	102.220
Postoperative	91.890	95.560	95.160
Pressure drop (*Δ*P, mmHg)	4.000	2.120	7.060
Peak WSS in LSA (Pa)*			
Preoperative	1.250	0.310	1.030
Postoperative	1.660	2.010	2.310
Change (*Δ*WSS, Pa)	+0.410	+1.700	+1.280
Peak Velocity in LSA (m/s)*			
Preoperative	0.450	0.280	0.470
Postoperative	0.590	0.480	0.700
Change (*Δ*Velocity, m/s)	+0.140	+0.200	+0.230
Flow Volume in LSA (ml/s)*			
Preoperative	37.030	34.110	32.270
Postoperative	31.290	18.230	9.710
Reduction (%)	−15.500	−46.600	−69.900
B. Mid-Diastole (80% of cardiac cycle)			
LSA Pressure (mmHg)*	45.430 → 45.390	57.540 → 57.530	57.360 → 57.380
Pressure drop (*Δ*P, mmHg)	0.040	0.010	−0.020
WSS in LSA (Pa)*	0.008 → 0.042	0.004 → 0.044	0.003 → 0.025
Velocity in LSA (m/s)*	0.019 → 0.063	0.011 → 0.043	0.004 → 0.013

LSA, left subclavian artery; WSS, wall shear stress.

*Values represent instantaneous measurements at two specific phases of the cardiac cycle: peak-systole (15% of the cardiac cycle) and mid-diastole (80% of the cardiac cycle), as defined by the inflow waveform (Figure 1B). No cycle-averaged values are presented; therefore, standard deviations are not applicable.*.

### Wall shear stress analysis

**Figure 5:**
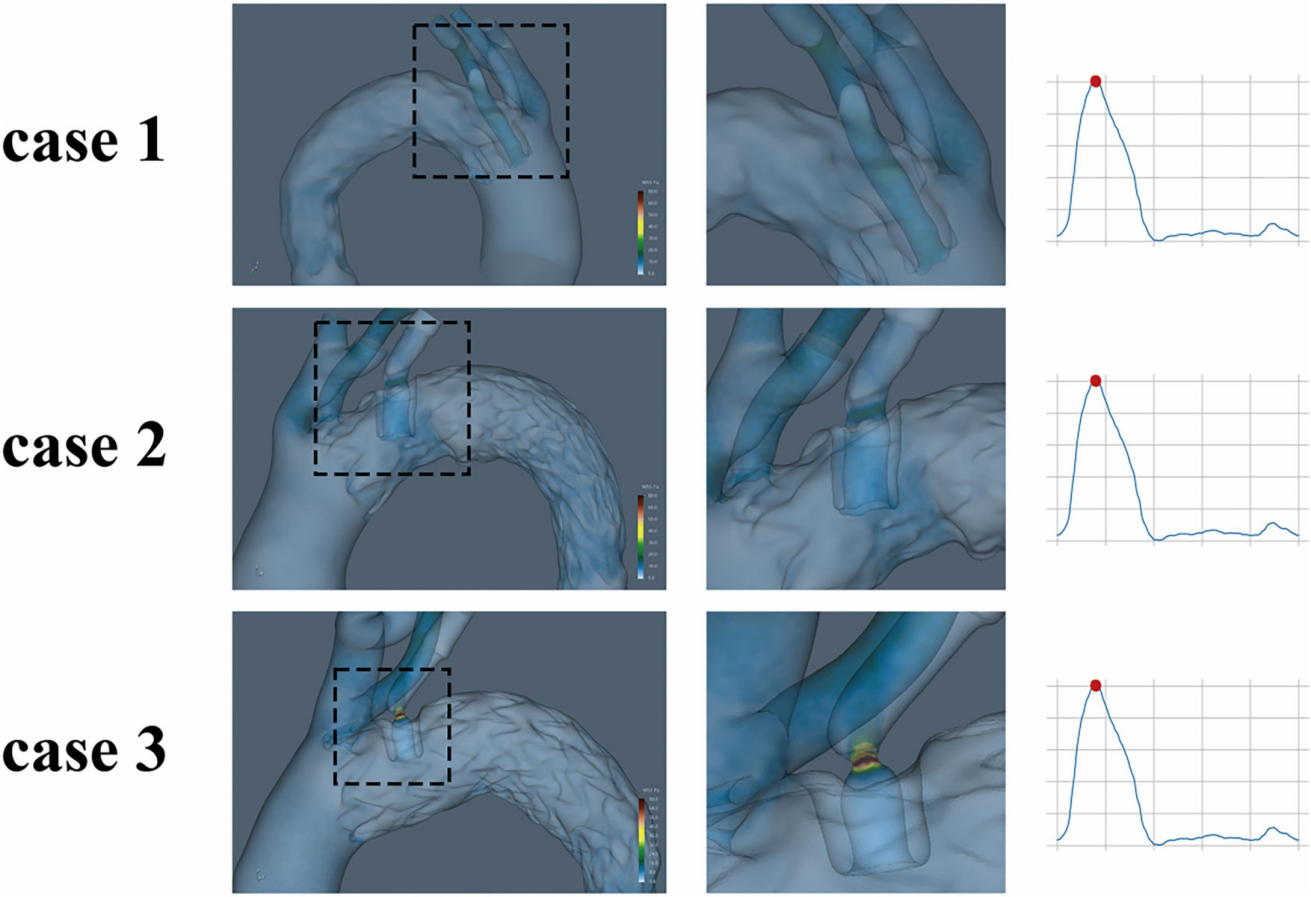
Wall shear stress-related indices distribution in the aortic arch after ISF-TEVAR.

WSS is a key hemodynamic parameter associated with risks of thrombosis and vessel injury. Figure 5 illustrates that WSS was elevated within the fenestrated stent segment of the LSA in all cases, particularly during systole. The magnitude of WSS increase correlated with the degree of stent compression. The highest postoperative peak WSS (2.31 Pa) was localized to the most compressed region in Case 3, whereas Case 1, with the least compression, presented the lowest peak WSS (1.66 Pa). Quantitative values are provided in Table 1.

### Blood flow characteristics

**Figure 6:**
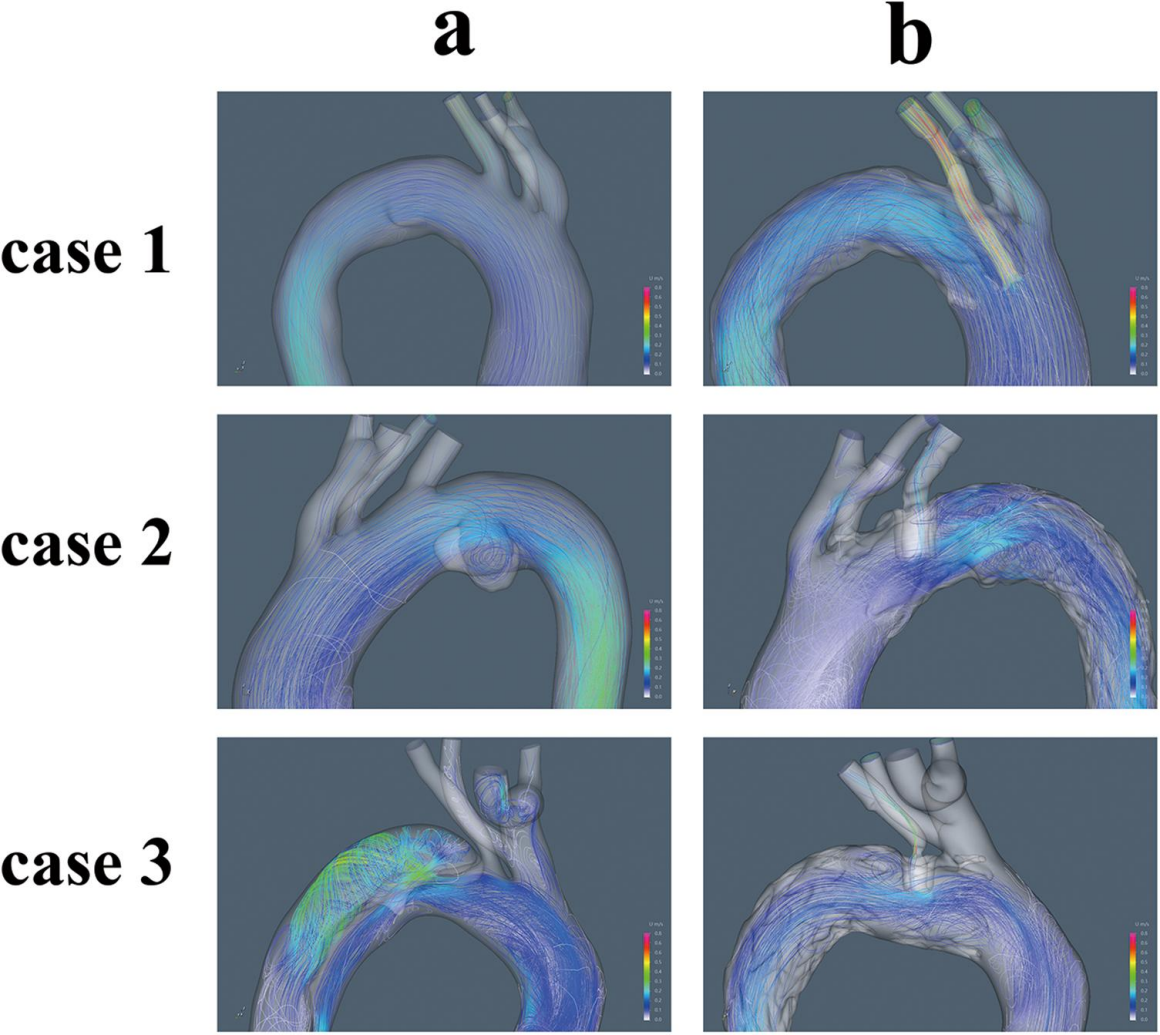
The distribution of blood flow velocity and turbulence in the aortic arch (**a**, pre-operation, **b**, post-operation).

**Figure 7:**
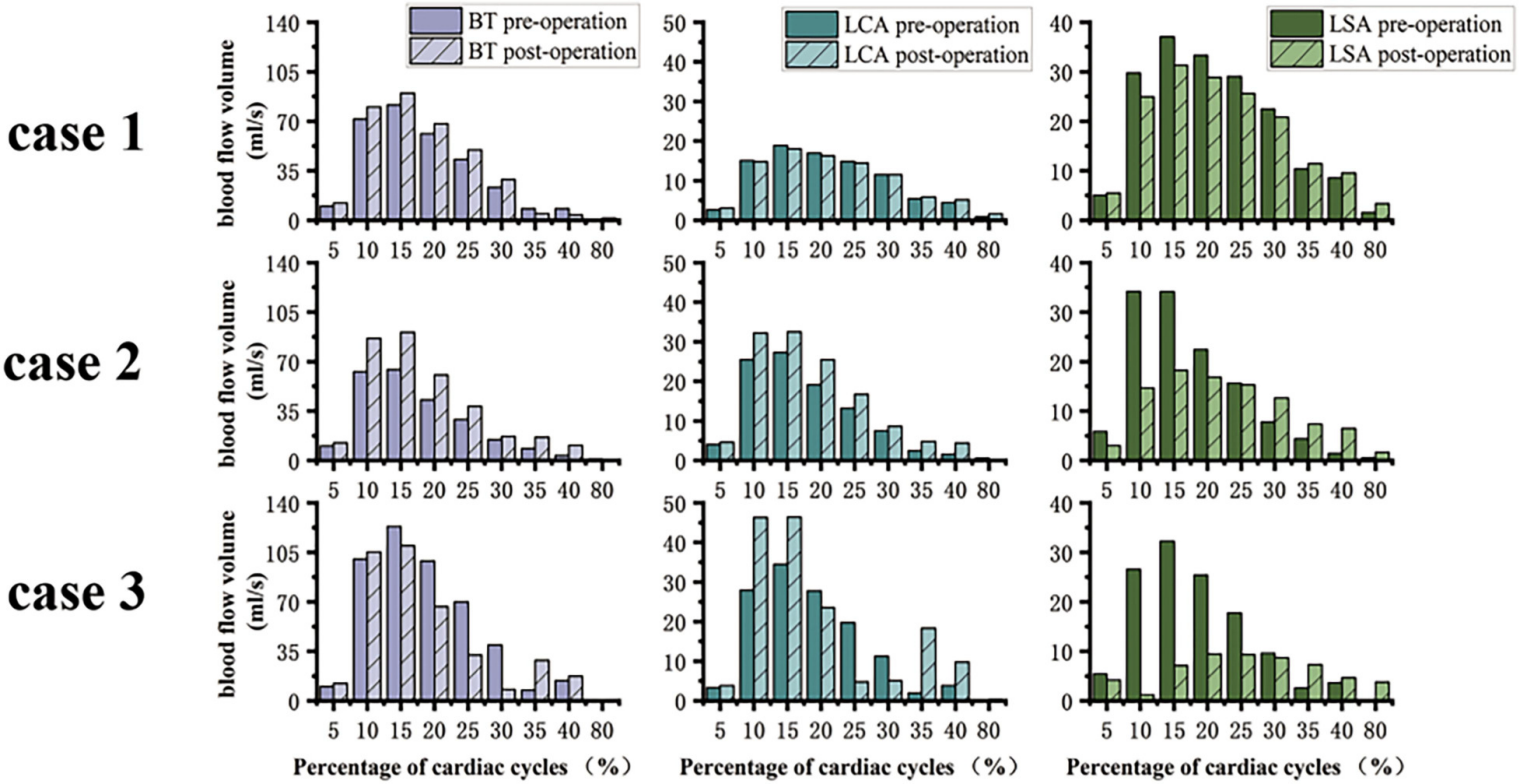
Changes in blood flow volume of the major branches of the aortic arch during a cardiac cycle. **(a)** pre-operation, **(b)** post-operation (BT, brachiocephalic trunk; LCA, left carotid artery; LSA, left subclavian artery).

Postoperative alterations in flow patterns were evident. Blood flow velocity within the LSA increased after ISF-TEVAR, with the highest peak systolic velocity (0.70 m/s) observed in Case 3 (Table 1). Flow visualization (Figure 6) revealed pronounced complex, disordered flow patterns. and vortex formation around the stent in Cases 2 and 3 during diastole, whereas flow remained relatively streamlined in Case 1. Concurrently, stent compression led to a significant reduction in LSA flow volume during systole. This reduction was most severe in Case 3 (−69.9%), corresponding to the highest compression rate, followed by Case 2 (−46.6%) and Case 1 (−15.5%), as quantified in Table 1 and shown in Figure 7.

### Comparison of CFD predictions with clinical outcomes

Despite the adverse hemodynamic parameters predicted by CFD—particularly in Case 3, which exhibited the highest compression ratio (58%), the greatest pressure drop (7.06 mmHg), the most substantial reduction in flow volume (−69.9%), and the highest peak WSS (2.31 Pa)—no patient developed clinical or imaging evidence of LSA insufficiency or thrombosis during the one-year follow-up period. All three patients remained asymptomatic with patent stents on CTA. This discordance between CFD-predicted risk and observed short-term clinical outcomes highlights that CFD identifies propensity for risk rather than predicting inevitable events, and underscores the protective role of antiplatelet therapy as well as the need for longer-term surveillance to determine whether these hemodynamic disturbances ultimately correlate with late complications.

## Discussion

In this patient-specific CFD study, we evaluated the hemodynamic consequences of different fenestration stent configurations following ISF-TEVAR. Our simulations revealed that postoperative hemodynamic alterations were predominantly localized to the LSA, with minimal changes observed in the other aortic arch branches.

Our study identified that the most significant hemodynamic alterations following ISF-TEVAR were localized to the left subclavian artery (LSA), with minimal changes observed in the other aortic arch branches. Quantitative analysis revealed that the degree of fenestration stent compression directly influenced distal perfusion in the LSA. Specifically, a higher compression ratio was associated with a more pronounced systolic pressure drop and a greater reduction in flow volume. This was most evident in Case 3, which exhibited the highest compression ratio (58%), the greatest pressure drop (7.06 mmHg), and the most substantial decrease in systolic flow compared to the other cases (24, 25). These findings gain clinical relevance in light of previous reports noting that stent occlusion following fenestration techniques can lead to upper limb ischemia (24, 25). Our analysis further demonstrated that stent compression, particularly in Case 3, was associated with significantly elevated wall shear stress (WSS) and increased flow velocity at the stenosis site. Given that elevated WSS is a well-established hemodynamic indicator for platelet activation and thrombus formation (26–29), the abnormally high WSS observed at the compressed region suggests a localized, heightened risk for thrombosis within the LSA stent. Therefore, to mitigate this potential risk, we suggest that during LSA reconstruction, every effort should be made to fully expand the fenestration stent to minimize compression, provided that endoleak is avoided. Additionally, careful stent deployment away from the reinforcing bars of the main stent graft may help prevent mechanical compression and its adverse hemodynamic consequences.

Stent configuration significantly influenced local flow patterns. Near-vertical insertion (*θ* = 85–90°, Cases 2 and 3) was associated with flow separation and prominent turbulence distal to the fenestration site, whereas the forward-leaning orientation (*θ* = 30°, Case 1) resulted in more physiologic, streamlined flow. Since highly disturbed flow is a known contributor to thrombogenesis (30–32), the choice of insertion angle emerges as an important modifiable factor affecting postoperative thrombotic risk.

All three patients underwent successful ISF-TEVAR and received standard dual antiplatelet therapy for six months postoperatively, followed by lifelong single antiplatelet therapy. At one-year follow-up, CTA imaging confirmed patent LSA stents in all cases, with no evidence of in-stent thrombosis, significant stenosis, or endoleak. Clinically, all patients remained asymptomatic, with no signs of upper limb ischemia or vertebrobasilar insufficiency.

Despite the adverse hemodynamic parameters predicted by CFD in Cases 2 and 3, particularly in Case 3, which exhibited the highest compression ratio (58%), the greatest pressure drop (7.06 mmHg), the most substantial reduction in flow volume (−69.9%), and the highest peak WSS (2.31 Pa). No patient developed clinical or imaging evidence of LSA insufficiency during the one-year follow-up period. Several factors may explain this apparent discordance between CFD-predicted risk and observed clinical outcomes. First, routine antiplatelet therapy likely provides effective protection against thrombus formation, even in the presence of hemodynamic disturbances known to promote platelet activation and aggregation (26–29). Second, the one-year follow-up duration may be insufficient to capture late thrombotic events; CFD-identified adverse hemodynamics represent a chronic pro-thrombotic milieu that may predispose to complications over longer timeframes. Third, it is important to distinguish between hemodynamic risk factors and clinical events—CFD identifies propensity for risk rather than predicting inevitable outcomes. The absence of short-term complications does not invalidate the relevance of these hemodynamic findings but rather underscores that thrombotic events likely require a combination of hemodynamic triggers, patient-specific factors, and sufficient time to manifest. This discrepancy highlights the importance of long-term surveillance beyond the initial postoperative period.

Importantly, the final stent configuration is heavily constrained by patient anatomy. A near-vertical insertion (*θ* = 85–90°) may be unavoidable when the LSA originates at a near-perpendicular angle from the aorta, as observed in Cases 2 and 3. To this end, we propose the following practical strategies. Preoperatively, the LSA takeoff angle should be carefully assessed on CTA to anticipate the likely stent configuration; cases with an acute takeoff angle (≥75° relative to the aortic arch centerline) should be recognized as having higher potential for hemodynamic disturbance. Intraoperatively, when vertical insertion is unavoidable, the fenestration should be created as close as possible to the LSA ostium to minimize insertion depth, as deeper insertion exacerbates compression at the fenestration interface. Post-dilation should be performed with a balloon diameter matching the nominal stent diameter to maximize expansion without over-dilation. Care should also be taken to avoid placing the fenestration directly over the reinforcing bars of the main stent graft, as this can create localized compression. Postoperatively, patients with vertical insertion and high compression ratios (e.g., >40%) may benefit from intensified surveillance, including Doppler ultrasound or CTA at six months and annually thereafter, along with strict adherence to dual antiplatelet therapy for at least six months followed by lifelong single antiplatelet therapy. These measures are particularly important given that CFD-identified adverse hemodynamics represent a chronic pro-thrombotic milieu that may predispose to late complications.

When anatomy permits a forward-leaning configuration, our data support opting for this orientation to achieve more physiologic flow patterns and reduce thrombotic risk. For patients with extremely challenging anatomy, such as an acute LSA take off combined with a narrow aortic arch or limited working space, alternative devices such as single-branch stent grafts (e.g., Castor) may offer a more favorable hemodynamic profile and should be considered during preoperative planning.

This study has several limitations. First, the small sample size, while suitable for a detailed, hypothesis-generating CFD analysis, limits the generalizability of the findings and prevents definitive conclusions. The hemodynamic outcomes are influenced by a confluence of factors including individual anatomy, which we cannot fully disaggregate in this small series. Second, the use of rigid-wall assumptions and Newtonian blood models, while common, may affect the accuracy of absolute WSS values, particularly in patients with differing vascular compliance. Future studies incorporating fluid-structure interaction and larger, multi-center cohorts are warranted to validate these preliminary observations and better elucidate the long-term clinical correlation of these hemodynamic parameters. Thirdly, the simulations were performed under a laminar flow assumption without incorporating a turbulence model. Although the Reynolds numbers in the aortic arch fell within the transitional range and this approach is acceptable for comparative analyses, it may not fully capture the turbulent kinetic energy or flow instability that could arise in regions with complex stent geometries, such as fenestration sites. Future studies employing large-eddy simulation (LES) or transition-sensitive models (e.g., SST *γ*–Re*θ*) may provide more accurate predictions of turbulence-related thrombogenic potential. Fourthly, the findings of this study are derived exclusively from self-expanding stent configurations (Ankura for the main stent graft and Viabahn for the fenestration branch). While the fundamental hemodynamic principles are universally applicable, such as the relationship between geometric compression and pressure drop, as well as the link between flow disturbance and wall shear stress, the quantitative thresholds and specific hemodynamic outcomes may differ for balloon-expandable stents (e.g., stainless steel or cobalt-chromium alloys), which are deployed by plastic deformation and possess different radial force characteristics. Therefore, the current findings should not be directly extrapolated to balloon-expandable platforms without further validation. Future studies incorporating both self-expanding and balloon-expandable devices are warranted to determine whether the observed hemodynamic benefits of minimizing compression and optimizing insertion angle apply consistently across different stent types. In addition, the use of rigid wall assumptions, while common in comparative CFD studies of aortic stenting, does not account for aortic compliance. Pulsatile wall motion may attenuate peak WSS and alter its spatial distribution, particularly at fenestration edges where flow disturbances are pronounced. Consequently, the absolute WSS values reported here may overestimate the true hemodynamic forces experienced *in vivo*, although the relative differences among stent configurations are expected to be preserved. Future studies incorporating FSI are warranted to more accurately quantify WSS magnitudes and to assess the impact of wall compliance on fenestration edge hemodynamics. Finally, while all patients remained free of clinical or imaging evidence of LSA insufficiency at one-year follow-up, the short follow-up duration may not capture late thrombotic events. The protective effect of routine antiplatelet therapy may also mitigate the thrombogenic potential of adverse hemodynamic conditions. Therefore, the long-term clinical significance of the hemodynamic differences identified in this study remains to be established in larger, prospective cohorts with extended surveillance.

## Conclusions

In this patient-specific CFD analysis of three ISF-TEVAR cases, we observed that high degrees of fenestration stent compression and a near-perpendicular insertion angle were associated with unfavorable hemodynamic alterations in the LSA, including significant pressure drops and elevated wall shear stress. These hemodynamic patterns are theoretically linked to increased thrombogenic potential. Therefore, during ISF-TEVAR, meticulous technique to minimize stent compression is crucial. When anatomy permits, a forward-leaning stent orientation may be hemodynamically preferable. For patients with unfavorable anatomy where vertical insertion is unavoidable, close postoperative surveillance and stringent antiplatelet therapy are emphasized. In such cases, alternative strategies like a single-branch stent graft (e.g., Castor) should be considered.

## Data Availability

The original contributions presented in the study are included in the article/Supplementary Material, further inquiries can be directed to the corresponding author/s.
